# Supplementary material for: Rational Design and In Vivo Characterization of mRNA-Encoded Broadly Neutralizing Antibody Combinations against HIV-1
Source: Antibodies (Basel). 2022 Oct 24;11(4):67. doi: 10.3390/antib11040067 (PMC9680504; doi:10.3390/antib11040067)
Supplement: Supplementary file 1 [file antibodies-11-00067-s001.zip › antibodies-1866574-supplementary.pdf]

Supplementary Information for: Rational Design and *in vivo* Characterization of mRNA-encoded Broadly Neutralizing Antibody Combinations Against HIV-1

Table S1. Neutralization antibody concentration inhibiting 80% of viral infection.

	IgG	scFv-Fc.r1	scFv-Fc.r2
		**PGDM1400**	
Mean (SD)	3.75 (5.4)	21.05 (53.67)	31.76 (90.05)
Median (IQR)	1.17 (0.13, 6.75)	3.05 (0.38, 7.72)	1.64 (0.32, 12.56)
		**10-1074**	
Mean (SD)	1.13 (2.46)	2.59 (7.45)	1.05 (2.14)
Median (IQR)	0.25 (0.07, 1.10)	0.14 (0.04, 0.45)	0.24 (0.07,0.91)
		**PGT121**	
Mean (SD)	3.67 (8.2)	2.72 (7.21)	2.54 (7.16)
Median (IQR)	0.13 (0.04, 0.56)	0.16 (0.07, 0.45)	0.12 (0.05, 0.42)
		**3BNC117**	
Mean (SD)	3.23 (7.43)	13.15 (11.08)	3.58 (3.37)
Median (IQR)	0.55 (0.17, 1.61)	11.09 (1.02, 25.00)	2.21 (0.71, 5.97)
		**N6**	
Mean (SD)	0.43 (0.51)		2.73 (3.00)
Median (IQR)	0.20 (0.05, 0.85)		1.33 (0.45, 5.24)
		**NIH45-46**	
Mean (SD)	0.38 (0.41)		2.73 (3.00)
Median (IQR)	0.24 (0.06, 0.75)		1.33 (0.45, 5.24)

IQR, interquartile range (25% percentile, 75% percentile); SD, standard deviation.

Table S2. scFv-Sc Round 2 Variant Mutations.

scFv-Sc Round 2 Variants	Mutations
**PGDM1400**	
Var_7	Linker20 (GGGGS) 4
Var_8	L20 + H:K23R
Var_9	L20 + H:K23R + H:K94F
Var_10	L20 + H:K19R
Var_11	L20 + H:K19R + S82AN
Var_12	L20 + S39Y
Var_13	VH_VL + L20
**10-1074**	
Var_7	Linker20 (GGGGS)4
Var_8	L20 + H:S79Y
Var_9	L20 + H:S19R
Var_10	L20 + H:K81Q
Var_11	L20 + H:S18R + H:S79Y + H:K81Q
Var_12	L20 + H:S18R + H:S79Y + H:K81Q +H:V68T
Var_13	L20 + H:L80Y
Var_14	L20 + H:S23Q
Var_15	L20 + H:S40R
Var_16	L20 + H:K13R
Var_17	L20 + H:K13R + S23Q
Var_18	VH_VL + L20

PGT121	
Var_7	Linker20 (GGGGS)4
Var_7a	L20 + 68N_Rev
Var_8	L20 + 68N_Rev + H:S79Y
Var_9	L20 + 68N_Rev + H:S19R
Var_10	L20 + 68N_Rev + H:S81Q
Var_11	L20 + 68N_Rev+ H:S19R + H:S79Y + H:S81Q
Var_12	L20 + H:S19R + H:S79Y + H:S81Q
Var_13	L20 + 68N_Rev + H:S81I
Var_14	L20 + 68N_Rev + H:S23Q
Var_15	L20 + 68N_Rev + L:S63M
Var_16	VH_VL + L20
**N6**	
Var_2	Linker20 (GGGGS)4
Var_3	L20 + H:A10Y
Var_4	L20 + H:K12R
Var_5	L20 + H:D81Q
Var_6	L20 + H:D81Q + H:R82AY

Var, variant.

Table S3. Kinetic–Pharmacodynamic Model Parameters.

**Parameter (unit)**	**Estimate**		
	**N6 IgG**	**PGT121 scFv-Fc Var7B**	**PGDM1400 scFv-Fc Var7**
m	0.862	0.566	0.438
CL_{ab} (mL/h)	0.00111	0.000719	0.000507
$t_{1/2}$ (days)	26.0	40.2	57.0

CL_{ab}, the antibody clearance rate from the central compartment; Fc, crystallizable fragment; IgG, immunoglobulin G; M, linear slope parameter; scFv, single-chain variable fragment; $t_{1/2}$, half-life.

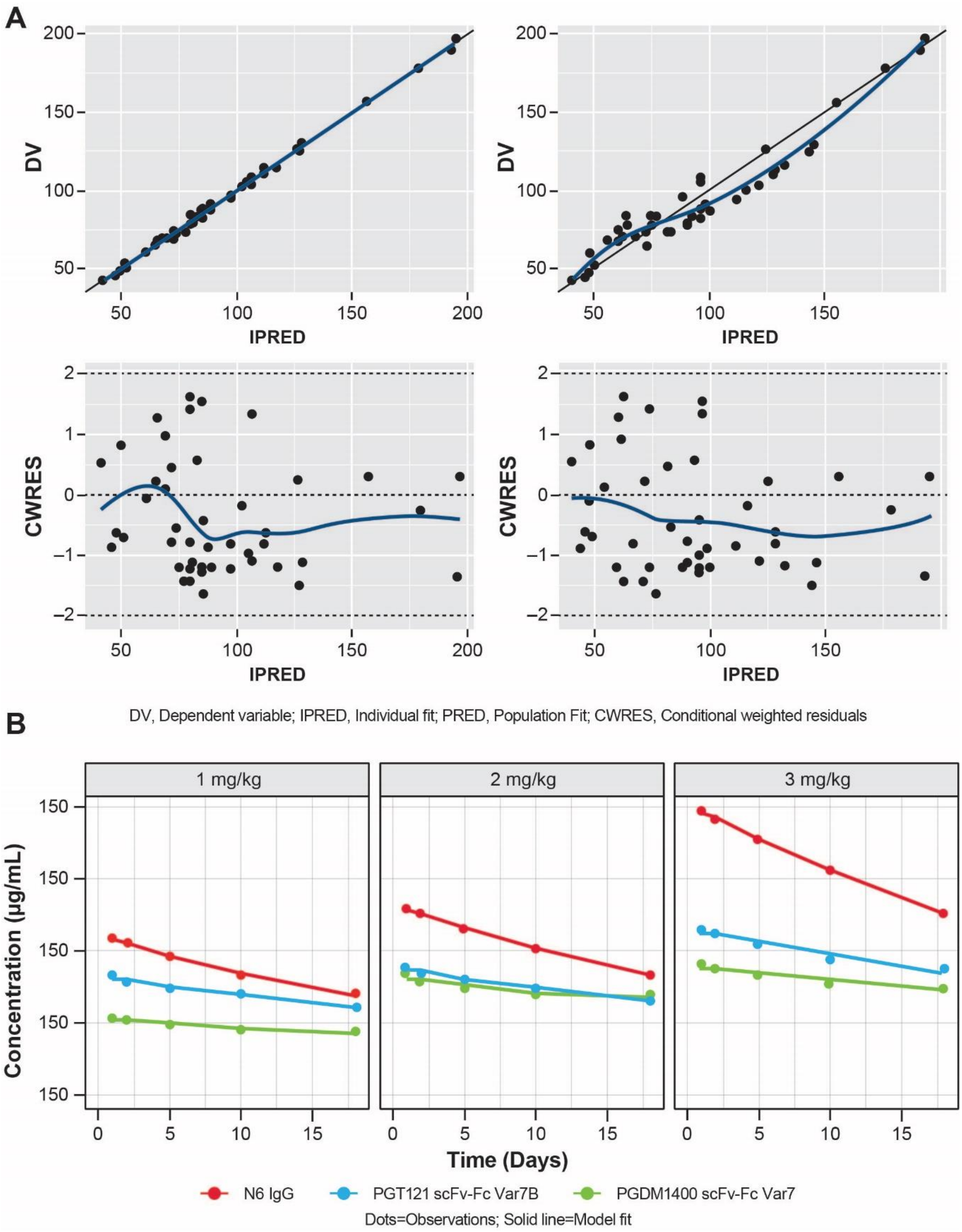

Figure S1. Kinetic–pharmacodynamic model diagnostics. (A) Goodness-of-fit plots and (B) fits for individual groups.
